# Sex differences in transcriptomic profiles in aged kidney cells of renin lineage

**DOI:** 10.18632/aging.101416

**Published:** 2018-04-18

**Authors:** Yuliang Wang, Diana G. Eng, Jeffrey W. Pippin, Sina A. Gharib, Aaron McClelland, Kenneth W. Gross, Stuart J. Shankland

**Affiliations:** 1 Paul G. Allen School of Computer Science and Engineering, University of Washington, Seattle, WA 98109, USA; 2 Division of Nephrology, University of Washington, Seattle, WA 98109, USA; 3 Department of Molecular and Cellular Biology, Roswell Park Cancer Institute, Buffalo, NY 14263, USA; 4 Institute for Stem Cell & Regenerative Medicine, University of Washington, Seattle, WA 98109, USA; 5 Computational Medicine Core, Center for Lung Biology, University of Washington, Seattle, WA 98109, USA

**Keywords:** glomerulus, podocyte, RNAseq, gene ontology, differentially expressed genes

## Abstract

Renin expressing cells in the kidney’s juxta-glomeruluar compartment likely also serve as progenitors for adult glomerular cells in disease. Although these cells of renin lineage (CoRL) decrease in number with advancing kidney age, accompanied by less responsiveness to typical stimuli such as ACE-inhibition, mechanisms and the impact of sex as a biological variable with age are not known. Accordingly, labeled CoRL were sorted from individual young (2m) and aged (27m) male and female Ren1cCre|ZsGreen reporter mice, and their transcriptomic profiles analyzed by RNA seq. When both aged female and male mice were combined, there were 48 differentially expressed genes (DEG) compared to young mice. However, when compared to their young sex-matched mice, aged female and male mice had 159 and 503 DEGs respectively. In addition to marked differences in individual genes between aged female and male mice, gene ontology analysis showed major pathway differences by sex. The majority of DEGs in one sex did not significantly change or changed in the opposite direction in the other sex. These results show that in CoRL of advanced age, individual genes and gene ontologies change, but differ between female and male mice, highlighting sex related differences the aging process.

## Introduction

With the global population living longer, increasing attention is focusing on the impact of advanced age on organ function, and how this might impact normal biological processes and pathways. Recent studies have shown functional and structural changes in the healthy aged kidney, and how these changes might impact outcomes in glomerular and tubulointerstitial diseases [1,2]. For example, because of their biological functions in blood pressure and sodium regulation, changes to the cells of renin lineage (CoRL) have characteristically been considered of major functional importance with increasing kidney age.

Healthy kidney aging is considered a hypo-reninemic state, based on lower plasma renin activity in human [3–15], and rats [16–20], reduced renin content in kidneys [16,17,20,21], and reduced responsiveness to certain stimuli known to increase renin [3–6,8,16,21,22]. Moreover, the number of CoRL decrease in mice with increasing age [21,23]. Yet, the precise mechanisms are not well understood.

Male and female sex impacts gene expression [24,25] and genomic stability [26]. Age associated changes to organs, and even age-related disease types, are also impacted by sex. For example, older men have higher rates of cardiovascular disease and Parkinson’s disease, while older women have more autoimmune disease and Alzheimer’s disease [27]. Females have a greater life span than males, which may or may not be X-linked and may also be due to longer telomeres in females [28]. Changes in sex steroids occur with age, which might for example underlie the greater prevalence of osteoporosis in older females, but better cardiovascular health compared to aged men. In the kidney, the age-related decline in kidney function is augmented in males [29–32], which is in part androgen dependent [33]. Even in non-aged animals, glomerular hemodynamics differ by sex [34]. There are also testosterone-mediated dimorphism for mouse proximal tubules [35,36]. For these and other reasons, increasing emphasis is being placed on the importance of sex as a biological variable in pre-clinical [37] and clinical studies [38].

Although we better understand how senescence [39], oxidative stress [40] and other pathways contribute to kidney aging, the precise mechanisms of age-related kidney changes remain poorly understood. Moreover, the impact of sex as a biological variable on kidney aging remains to be elucidated. The purpose of the current study was to better understand the impact of age and sex on potential pathways associated with aging of cells of renin lineage. Accordingly, we isolated cells of renin lineage from young and aged mice from both sexes, then quantitated and compared their transcriptomic profiles measured by RNA-seq.

## RESULTS

### Effect of age on the global transcriptomic changes in cells of renin lineage

**Figure 1:**
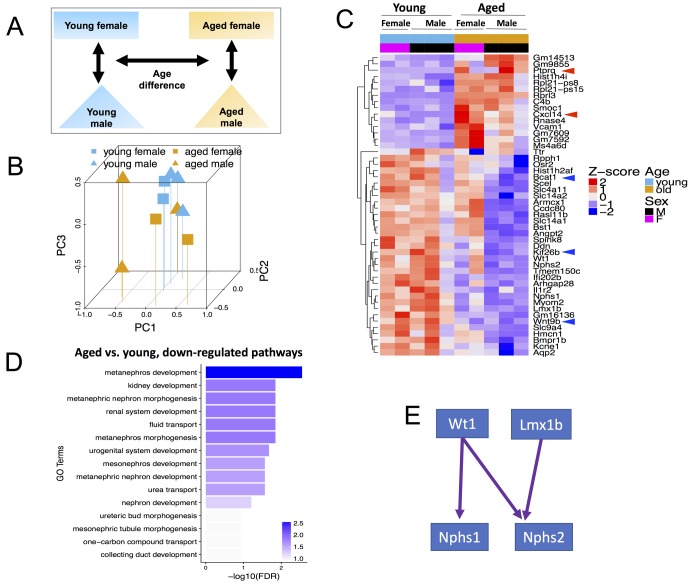
**Global transcriptome changes associated with CoRL aging when both male and female mice are included in the analysis.** (**A**) Schema showing comparisons made included age and both sexes. (**B**) Principal Component Analysis (PCA) plot of all (n=10) RNA-seq samples. PC1 represents the largest variation in gene expression, followed by PC2, then PC3. Blue and mustard colors denote young and aged mice respectively; squares and triangles denote female and male sex respectively. Young female, young male, aged female and aged male samples mostly tend to cluster together. (**C**) Heat map of all samples showing clusters of genes with consistent changes in aged male and aged female mice compared to young sex counterparts. The Z-score colors are shown, with blue indicating genes with lower than overall mean expression levels, and red indicating genes with higher than mean expression levels. (**D**) In aged female and male mice, clustering by Gene Ontology (GO) terms identified the consistently down-regulated genes as metanephros development, kidney development and metaneprhic nephron morphogenesis. (**E**) The developmental transcription factors, Wt1 and Lmx1b, and their key target genes, nephrin (Nphs1) and podocin (Nphs2) were down-regulated in aging.

We hypothesized that with advancing age, cells of renin lineage (CoRL) have reduced stemness, accompanied by a reduced ability to proliferate and migrate from the juxta-glomerular compartment to the intra-glomerular compartment, with increased cell death compared to young mice. To identify key genes that may regulate these processes, we performed RNA-seq on 5 young (2 months) CoRL reporter mice (3 male, 2 female) and on 5 aged (27 months) inducible CoRL reporter mice (3 male, 2 female) (Fig. 1A).

To begin our analysis, we first reduced our high dimensional dataset, being RNA expression levels for each gene, into fewer dimensions in order to identify if there were systematic differences in our four types of samples. We performed principal component analysis (PCA) to identify the dimension that explains the most variance in our data set (PC1) and the dimensions that explain the remaining variances (PC2, PC3, etc.). The PCA plot in Figure 1B shows that both sex and age contributed to global transcriptomic variation for cells of renin lineage.

48 genes (FDR<0.01, Figure 1C and Table S1) were identified with significant consistent differences between aged and young mice of both sexes. Of these differentially expressed genes (DEGs), 15 genes were up-regulated in aged mice (Z score 0-2), and 33 were down-regulated with age compared to young mice (Z score -2-0). Because CoRL serve as adult progenitors for mesangial cells [41], glomerular parietal epithelial cells [42], podocytes [42–45] and pericytes [46], we overlapped these 48 DEGs (Figure 1C) with known pluripotency genes, defined as those highly expressed in human embryonic stem cells (hESC), down-regulated in differentiation and expression in somatic tissue is <5% of hESC expression [47] and found Bcat1 (Branched Chain Amino Acid Transaminase 1) [48–51] a target gene of kidney stem cells modulator c-Myc [50,52]. Other downregulated genes involved in cell proliferation and migration include Wnt Family Member 9B (Wnt9b) [53] and Kinesin Family Member 26B (Kif26b) [54].

Age-related up-regulated DEGs important in cell proliferation, migration and apoptosis include C-X-C Motif Chemokine Ligand 14 (Cxcl14) [55], and Protein Tyrosine Phosphatase, Receptor Type Q (Ptprq) [56], which is increased in renal injury [57] Taken together, both down- and up-regulated genes in aged CoRL was consistent with a decline in stemness, migration, proliferation, and an increase in apoptosis, supporting the notion that with advancing age, their pluripotent stemness decreases.

We next performed Gene Ontology (GO) term enrichment analysis and found significant enrichment for multiple kidney development and function GO terms among down-regulated DEGs, such as metanephros development (e.g., Wt1, Wnt9b) and fluid transport (e.g., Slc4a11, Slc14a1) (Figure 1D). Among the down-regulated genes, we focused on four genes, Wt1, Nphs1, Nphs2 and Lmx1b, with important roles in kidney development and podocyte function. These genes form a transcriptional regulatory network, where Wt1 regulates nephrin (Nphs1) [58] and podocin (Nphs2) [59], and Lmx1b regulates podocin [60] (Figure 1E). Coherent down-regulation of these four genes suggests that they may play an important role in CoRL aging, and perhaps help explain their reduced capacity to act as podocyte progenitors with age.

### Transcriptomic changes in aged female and male cells of renin lineage

**Figure 2:**
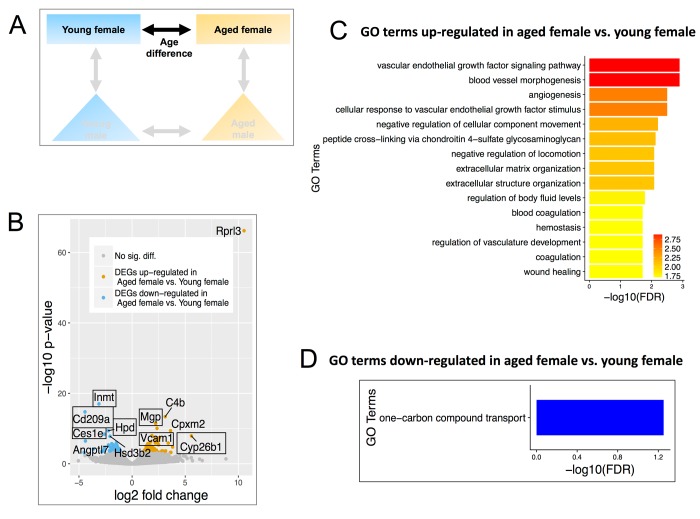
**Differentially expressed genes (DEGs) and enriched Gene Ontology (GO) terms in aged female vs. young female.** (**A**) Comparisons include age and females only. (**B**) Volcano plot showing upregulated (mustard) and down-regulated (blue) DEGs with a minimum 1.5-fold change and a FDR <0.1. Text boxes highlight individual genes discussed in results section. (**C**) Gene ontology terms significantly enriched in the genes found to be significantly upregulated in aged female vs. young female mice. Many of these GO terms are related to endothelial cell signaling and maintenance. (**D**) GO terms of down-regulated genes in aged females.

The global transcriptome analysis in Figure 1 demonstrates that even among genes that show the same trend in both aged female and male mice compared to both young female and male mice, there are obvious differences in gene expression between sexes within the same age groups (i.e., within aged and young). Accordingly, we compared aged versus young CoRL within each sex to identify sex-specific changes in the aging process in this cell type (Figure 2).

#### Aged vs. young female mice

We first analyzed differentially expressed genes (DEGs) between aged versus young female mice (Figure 2A**)**. Using cutoffs as FDR<0.1 and a fold change >1.5, 159 DEGs were identified, of which 95 were up-regulated in aged and 64 were down-regulated in aged (Figure. 2B, Table S2). Volcano plots in aged female mice showed increases in calcification inhibitor matrix Gla protein (Mgp), vascular cell adhesion molecule 1 (Vcam-1), and retinoic acid-degradation enzyme Cyp26b1. Mgp is known to be enriched in adult renin cells [61]. Several genes decreased in aged female mice compared to young female mice, including those involved in xenobiotic metabolism (Inmt, Hpd, Ces1e) and Cluster of Differentiation 209 (Cd209a) (aka. Dendritic Cell-Specific Intercellular adhesion molecule-3-Grabbing Non-integrin, DC-SIGN) (Figure 2B), which co-expresses with nephrin in podocytes [62].

Up-regulated genes in aged female CoRL were significantly enriched for GO terms related to angiogenesis and ECM organization, such as Flt4, PDGF Rec A, VEGFd (Figure 2C). One-carbon compound transport was the only GO term for down-regulated genes (Figure 2D).

#### Aged vs. young male mice

**Figure 3:**
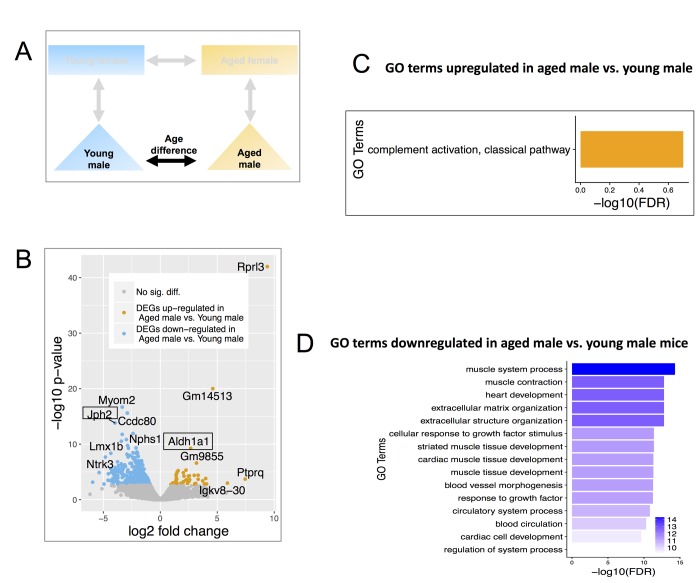
**Differentially expressed genes (DEGs) and enriched GO terms in aged male vs. young male.** (**A**) Comparisons include age and males only. (**B**) Volcano plot showing upregulated (mustard) and down-regulated (blue) DEGs with a minimum 1.5-fold change and a FDR <0.1. Text boxes highlight individual genes discussed in results section. (**C**) Gene ontology terms significantly enriched in genes significantly up-regulated in aged male vs. young male. Many of these GO terms are related to immune responses and differ from those enriched in aged female vs young female mice. (**D**) Significantly enriched Gene Ontology terms down-regulated genes in aged male vs. young male. These genes are interestingly related to muscles and were also not identified as enriched in aged female vs young female mice. The results are consistent with the notion of distinct differences in the aging process between males and females.

When comparing aged versus young male gene expression profiles (Figure 3A), 503 DEGs were identified using the same cutoffs as above, of which 52 were up-regulated in aged males, and 451 were down-regulated (Figure 3B, Table S3). Compared to young male mice, retinoic acid-synthesis enzyme Aldh1a1 was up-regulated in aged male mice. Aldh1a1 has previously been shown to be significantly up-regulated in aged vs. young *females* using whole kidney samples [63]. Junctophilin 2 (Jph2), a gene related to Ca2+ control of renin synthesis and release previously shown to be highly expressed in CoRL [61] was down-regulated in aged male mice (Figure 3B).

Up-regulated genes in aged males were significantly enriched in immune responses (e.g., complement 1 a, b, c, Figure 3C), while genes down-regulated were enriched for GO terms relating to muscle contraction & development (e.g. myomesin 2, myocardin, Figure 3D).

### CoRL aging dynamics exhibit sex dimorphism

We found that CoRL aging dynamics differs significantly between female and male based on the following observations. First, transcriptomic perturbation is much larger in aged males than in aged females, based on the number of DEGs (503 in male vs. 159 in female, binomial proportions test p-value <2.2x10^-16^). This difference is not due to larger sample size for males (3 vs. 3 in male; 2 vs. 2 in female): after randomly removing one male sample from each age group, and recalculating DEG, we still observed more DEGs (n=478) in male than female. Second, genes perturbed in the female aging process behave differently from male aging, and vice versa. For example, Aldh1a1 was up-regulated in male CoRL but not in female CoRL. Myom2, Nphs1, Lmx1b showed greater reduction in aged males vs. young males than in aged females vs. young females.

**Figure 4:**
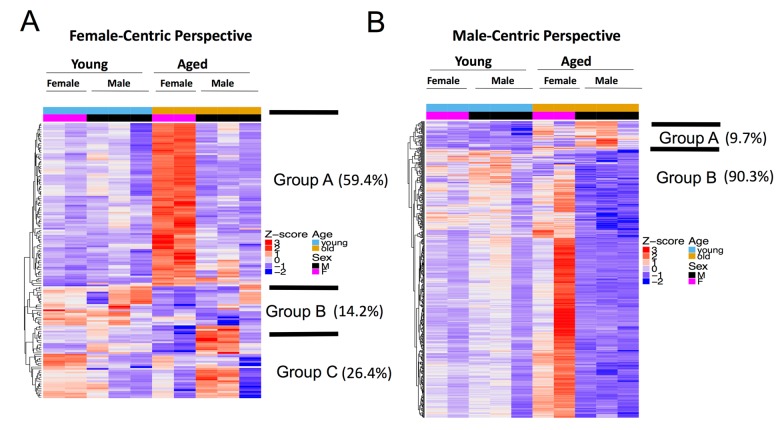
**Female and male aging gene dynamics are different.** (**A**) *Female-centric perspective*. Expression pattern of aged female vs. young female DEGs (n=159) across all age and sex conditions. Group A genes (comprises 59.4% of all DEGs) are significantly up-regulated in aged vs. young female, but do not have any significant change in aged vs. young male. Group B genes decreased in both aged vs. young female and aged vs. young male. Group C genes show the opposite trend, as they are down-regulated in aged vs. young female, but up-regulated in aged vs. young male. (**B**) *Male-centric perspective.* Expression pattern of aged male vs. young male DEGs (n=503) across all age and sex conditions. Group A genes (comprises 9.7% of all DEGs) are up-regulated in aged vs. young male and are also moderately up-regulated in aged vs. young female. Group B genes are significantly down-regulated in aged vs. young male are up-regulated in aged female mice.

We therefore took a female-centric view of the aging process and examined the expression pattern of aged versus young female DEGs (excluding genes in X and Y chromosomes) across all sex and age groups and found three distinct groups (Figure 4A). Genes in group A (92 out of 155 autosomal DEGs, 59.4%), enriched for blood vessel morphogenesis and extracellular matrix organization GO terms, are significantly up-regulated in aged vs. young female, but do not have any significant change in aged vs. young male. Genes in group B (22 out of 155, 14.2%) (e.g., Nphs1, Myom2 and urea/CO2 transporters) decreased in both aged vs. young female and aged vs. young male. Genes in group C (41 out of 155, 26.4%) show the opposite trend: they are down-regulated in aged vs. young female, but up-regulated in aged vs. young male.

We then took a male-centric view and examined the profile of DEGs in the male aging process across all samples. Genes up-regulated in aged vs. young male are also moderately up-regulated in aged vs. young female (group A, 47 out of 487 autosomal DEGs, 9.7%, Figure 4B). However, most of the DEGs that were significantly *down*-regulated in aged vs. young males were *up*-regulated in aged female mice (440 out of 487, 90.3%, group B, Figure 4B). Perturbed pathways during the aging process are also different between female and male mice (Figure 2B, 2C, 3B and 3C). It is clear that genes perturbed in the aging process are distinct between males and females, i.e., transcriptome perturbations in the aging process exhibit sex dimorphism.

### Sex differences in CoRL increases with aging

**Figure 5:**
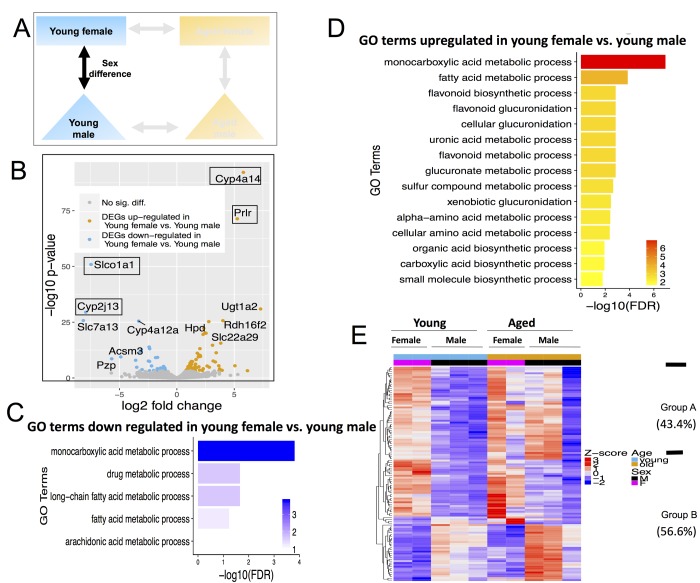
**Differentially expressed genes (DEGs) and pathways in young female vs. young male.** (**A**) Comparisons made included age with female and male-centric views. (**B**) Volcano plot showing up-regulated (mustard) and down-regulated (blue) DEGs, and those not significant (grey). Text boxes highlight individual genes discussed in results section. (**C**) Gene ontology (GO) terms significantly enriched in genes up-regulated in young female vs. young male mice. (**D**) GO terms significantly enriched in genes down-regulated in young female vs. young male mice. (**E**) Expression pattern of young female vs. young male DEGs across all age and sex conditions. Group A genes (43.4% of all DEGs) are decreased with age; Group B genes (56.6%) either persisted or increased with age.

In light of sex-specific transcriptomic changes in the aging process, we next compared male and female samples within each age group and examined how such sex differences change with age (Figure 5A).

#### Young female vs. young male mice

There were 113 differentially expressed genes between young females vs. young males. Of these DEGs, 79 were up-regulated in females and 34 were down-regulated in females (Figure 5A, Table S4) The most up-regulated genes in young females were cytochrome P450, family 4, subfamily a, polypeptide 14 (Cyp4a14) and prolactin receptor (Prlr). The most down-regulated genes are solute carrier organic anion transporter family, member 1a1 (Slco1a1) and Cytochrome P450, family 2, subfamily j, polypeptide 13 (Cyp2j13) (Figure 5B).

Interestingly, both the up- and down- DEGs in young female vs. young male mice were enriched for monocarboxylic acid (which includes fatty acid) and xenobiotic metabolic processes (Figure 5C and 5D). When clustered based on their expression profile across all sex and age groups, these 113 DEGs fall into two distinct categories. Sex differences in group A (49 out of 113, 43.4%) decreases with age while differences in group B (64 out of 113, 56.6%) persisted or increased with age (Figure 5E). In particular, genes expressed higher in young male compared to young female mice are expressed even higher in aged male than in aged female, consistent with these differences being even more pronounced with advanced age (Figure 5E).

#### Aged male vs. aged female mice

**Figure 6:**
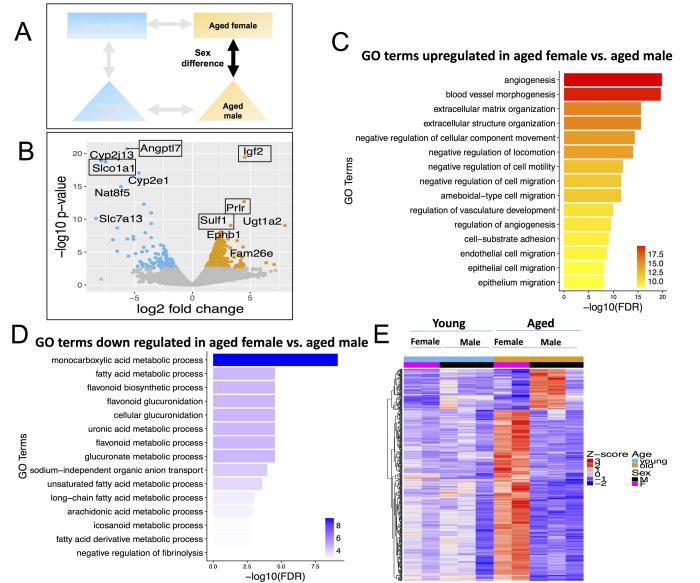
**Differentially expressed genes (DEGs) and pathways in aged female vs. aged male.** (**A**) Comparisons made included sexes between same aged groups. (**B**) Volcano plot of differentially expressed genes; (**C**) Gene ontology terms significantly enriched in genes up-regulated in aged female vs. aged male. (**D**) Gene ontology terms significantly enriched in genes down-regulated in aged female vs. aged male. (**E**) Expression pattern of aged female vs. aged male differential genes across all age and sex conditions.

Finally, there were 546 DEGs in aged females vs. males (Figure 6B, Table S5), which is 4.8-fold more DEGs than in young female vs. male (113 DEGs). Of these, 441 were up-regulated in aged females and 105 were down-regulated in aged females. When comparing aged female and aged male to one another, the major genes that increased in aged female were Insulin Like Growth Factor 2 (Igf2), Prolactin Receptor (Prlr) and Sulfatase 1 (Sulf1) (Figure 6A). Igf2 is known to regulate adult hematopoetic stem cell activity [64] and Sulf1expression decreases proliferation and migration in cancer cell lines [65]. The major genes downregulated were Angiopoietin Like 7 (Angptl7) and solute carrier organic anion transporter family, member 1a1 (Slco1a1) (Figure 6A).

Up-regulated DEGs in aged females were enriched for angiogenesis (Figure 6C); while down-regulated DEGs in aged females (higher in aged male) were again enriched for fatty acid metabolism (Figure 6D). Only 35 genes are differentially expressed between female vs. male in both young and aged age groups. Therefore, 96% of the differentially expressed genes in aged female vs. aged male mice arise *de novo* in the aging process, i.e., they are *not* differentially expressed in young female vs. young male (Figure 6E).

### Gene Set Enrichment Analysis of CoRL aging

**Figure 7:**
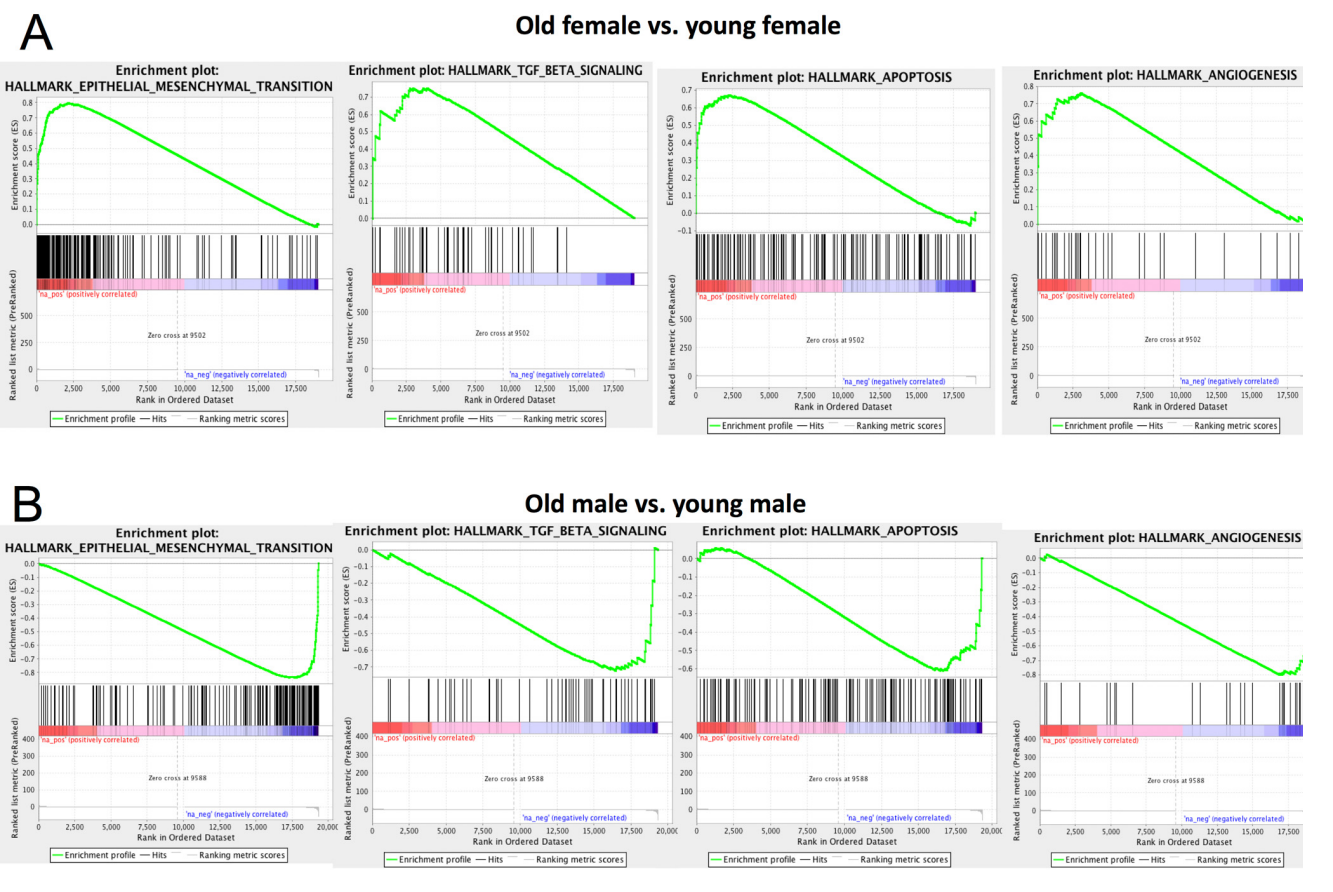
**Gene Set Enrichment Analysis (GSEA).** GSEA results for four pathways in old vs. young female (**A**) and old vs. young male (**B**). These four pathways show opposite change in female aging compared to male. Full GSEA results are reported in Table S6, Table S7, Table S8, Table S9.

We performed Gene Set Enrichment Analysis [66] using the Hallmark gene set [67] for both aged vs. young female and aged vs. young male mice (Table S6-9). We observed that epithelial-mesenchymal transition, TGF β, apoptosis and angiogenesis pathways are significantly up-regulated in aged females compared to young females (Figure 7A). This is consistent with previous results that these pathways play important roles in CoRL aging [46,68,69]. Surprisingly, these pathways are down-regulated in aged male vs. young male (Figure 7B), further supporting that CoRL aging process is different between male and female. In particular, multiple matrix metallopeptidase genes (e.g., Mmp2, Mmp3, Mmp14) that regulate EMT [70–72] are significantly up-regulated in aged female but not aged male. Down-regulation of biglycan (Bgn) is known to inhibit cell cycle and induce apoptosis [73]. Interestingly, biglycan is significantly up-regulated in aged female but down-regulated in male.

## DISCUSSION

Recent studies have shown that in addition to cells of renin lineage (CoRL) serving a major endocrine function, they likely also have a major biological role serving as progenitors for adult mesangial cells, podocytes, glomerular parietal epithelial cells and pericytes [23,41–44,68]. Advanced aging is accompanied by reduced renin content in CoRL [3,10,74], and decreased CoRL number in aged mice [23]. In addition, CoRL proliferation in aged mice is blunted in response to ACE-inhibitors [21]. Because gene expression in CoRL might provide insights into potential mechanisms for changes during aging, we performed RNAseq on sorted CoRL to define changes in their transcriptional profile in aged mice compared to young age mice. Overall sex also had a major impact in gene patterns with advanced age.

When the results from both female and male mice were combined in to the overall analysis, there were only 48 differentially expressed genes in Ren1cCre|ZsGreen mice aged 27 months (equivalent to human aged 79 years) compared to combined young female and male mice aged 2 months (equivalent to young human adult). However, the first major finding was that when aged female mice and aged male mice were compared to their young sex matched counterparts separately, the number of differentially expressed genes (DEGs) was significantly higher than when analyzed together. Compared to young sex matched mice, aged female mice had 159 DEGs, and aged males had 503 DEGs.

The second major finding was that within age-associated DEGs, the genes and pathways differed substantially between aged female and aged male mice over sex-matched young mice. In addition to marked differences in individual genes between aged female and male mice, gene ontology analysis showed major pathway differences by sex. Aged females had marked increases in vascular endothelial growth factor signaling, blood vessel morphogenesis and extracellular matrix organization in CoRL, whereas aged males had increases in immune and inflammatory pathways such as complement activation, immunoglobulin immune response, B cell immunity and humoral response. Downregulated pathways also differed by sex in aged mice compared to young counterparts. In aged females, one-carbon compound (e.g., CO2, urea) transport decreased, whereas muscle system processes and contraction, heart development and extracellular matrix and structure organization were decreased in aged males. Consistent with the observation that the progression of chronic kidney disease is faster in males [75], these results showed larger transcriptomic perturbations in aged males compared to aged females (503 DEGs in male, 159 in female, binomial proportions test p-value <2.2x10^-16^). In addition to the magnitude of transcriptomic perturbations, perturbed genes show very different dynamic patterns in the aging process of different sexes: the majority of DEGs in one sex do not significantly change or change in the opposite direction in the other sex. Thus, it is critical to include samples/subjects from both sexes when studying kidney aging.

The third major finding was that specific patterns emerged within the DEGs when comparing sex and age. From a female perspective, three groups emerged. The largest was group A, enriched for blood vessel morphogenesis and extracellular matrix organization genes which increased in aged female mice but not aged male mice. Group B genes (e.g., Nphs1, Myom2 and urea/CO2 transporters) decreased in both aged female and male mice compared to their young controls, whereas group C genes were decreased in aged female but increased in aged male. From a male-centric view, of the 503 DEGs, group A genes were increased in both aged male and female mice, while group B, enriched for muscle contraction and blood morphogenesis genes were down-regulated in aged male mice, yet increased in aged female mice. We also found that nitric oxide synthase (NOS) expression decreased in aged vs. young male, but not in aged vs. young female (Table S2 and Table S3). While previous studies have reported sex dimorphism in NOS activity in kidney aging [76], our results showed much more widespread sex differences in the kidney aging process.

We recognize some limitations in these studies. First, animal number was limited. However, this exploratory approach should be followed up by more definitive studies on candidate genes pertaining to their role in CoRL aging. Second, only one aged time point was measured, limiting a full interpretation of when changes in transcriptomic profiles occurred. Third, the exact location within the kidney of the FACS sorted cells of renin lineage was not specified, although the majority are typically in the juxta-glomerular compartment. Fourth, we acknowledge that although we used FACS sorting to isolate CoRL based on GFP, the gene de-enrichment results might be due to a small amount of contamination from tubular epithelial cells.

In summary, we found a small core set of genes involved in stemness, proliferation and migration that showed consistent changes in CoRL aging. Surprisingly, most gene expression changes in CoRL aging are sex-specific: aged female mice showed up-regulation of epithelial to mesenchymal transition, angiogenesis, and TGFβ signaling, while male mice showed opposite change in these pathways. Follow up studies are needed to prove the biological roles of these genes in aged kidneys.

## METHODS

### Animals

Ren1cCre|ZsGreen mice as previously described [23] were bred and maintained in house and allowed to age for either 2 months (n = 5, 3M, 2F) or 27 months (n = 5, 3M, 2F) prior to sacrifice. These mice are the product of breeding the Ren1cCre mouse [77] with the ZsGreen mouse [78], and results in the constitutive and permanent labeling of all cells that have expressed renin. Mice were housed, provided ad lib chow and water, in the animal care facility of the University of Washington, under specific pathogen-free conditions. Studies were reviewed and approved by the University of Washington Institutional Animal Care and Use Committee (2968-04).

### Isolation of cells of renin lineage for RNA sequencing

At the ages stated above, animals were sacrificed, the kidneys were removed and dissected free of fat and the kidney capsule. Kidney cortex was digested in 0.2mg/ml Liberase™ TL (Sigma-Aldrich, St. Louis, MO), 100 U/ml DNAse I (Sigma-Aldrich, St. Louis, MO) in RPMI 1640 medium, without L-glutamine or phenol red (GE Healthcare Bio-Sciences, Pittsburgh, PA) by shaking in a 37^o^C water bath for 30 minutes. The digest was passed through a 22G needle (Becton Dickenson, Franklin Lakes, NJ) 10 times to further dissociate the tissue, then inactivated by combining with 5ml of in media consisting of RPMI 1640 medium, without L-glutamine or phenol red (GE Healthcare Bio-Sciences, Pittsburgh, PA) supplemented with 1mM sodium pyruvate (ThermoFisher Scientific, Waltham, MA), 9% Nu-Serum™ IV Growth Medium Supplement (Corning Incorporated - Life Sciences, Durham, NC) and 100U/ml Penicillin-Streptomycin (ThermoFisher Scientific, Waltham, MA). The suspension was passed through a 100µm, then a 40 µm cell strainer (BD Biosciences, San Jose, CA), to clear multicellular debris, then pelleted by centrifugation at 200G at 4^o^C for 5 minutes. The cells were re-suspended in the media described above, counted and isolated using multicolor fluorescence-activated cell sorting (FACS) on a BD FACS Aria II (BD Biosciences, San Jose, CA) housed within a BSL1/2 approved biosafety cabinet. Sorted cells were pelleted by centrifugation and snap frozen in liquid nitrogen until isolation of RNA. The entire procedure took 6-7h per animal. Samples were then shipped on dry ice to Roswell Park Cancer Institute for further processing.

### RNA isolation

The purification of total and RNA was performed using the miRNeasy micro kit (Qiagen, Germantown, MD). Flow sorted cells were homogenized with the addition of 700 ul of QIAzol Lysis Reagent. After addition of chloroform, the homogenate was separated into aqueous and organic phases by centrifugation. The upper, aqueous phase was extracted. On-column DNAse digestion was also performed to remove any residual genomic DNA contamination followed by additional washes. High quality RNA was then eluted in 14 ul of RNase-free water. Quantitative assessment of the purified total RNA was accomplished by using a Qubit Broad Range RNA kit (Thermo Fisher, Waltham, MA). The RNA was further evaluated qualitatively by a 2100 Bioanalyzer (Agilent Technologies, Santa Clara, CA).

### RNAseq library preparation and sequencing

Amplified cDNA was generated using the SMART-Seq v4 Ultra Low Input RNA kit (Clontech, Mountain View, CA). Total RNA (10 ng) was used to synthesize first-strand cDNA utilizing proprietary template switching oligos. Amplified double stranded cDNA is created by LD PCR using blocked PCR primers and unique sample barcodes were incorporated. The resulting cDNA was purified using AmpureXP beads (Beckman Coulter, Brea, CA). Abundant Ribosomal cDNA was then depleted using R probes and 12 cycles of PCR with universal PCR primers completed the library. The final libraries were purified using AmpureXP beads, and validated for appropriate size on a 4200 TapeStation D1000 Screentape (Agilent Technologies,, Santa Clara, CA). The DNA libraries were quantitated using KAPA Biosystems qPCR kit and pooled together in an equimolar fashion. Each pool was denatured and diluted to 16pM for On-Board Cluster Generation and sequencing on a HiSeq2500 sequencer for 100 paired-end sequencing following the manufacturer’s recommended protocol (Illumina Inc., San Diego, CA).

### RNAseq data analysis

RNA-seq were aligned to mm9/NCBIM37 using Tophat (version 2.0.13) [79]. Gene-level read counts were quantified using featureCounts [80] using Ensembl NCBIM37 gene annotations. *prcomp* function from R was used to for Principal Component Analysis.DESeq [81] was used for differential gene expression analysis. Two models were built to find genes that change consistently between sexes during aging. The null model assumed gene expression variation was purely due to sex differences; the full model additionally included aged vs. young as a predictor. A gene was identified as significant when the full model fit was better (Benjamini-Hochberg FDR<0.1). topGO R package [82] was used for Gene Ontology enrichment analysis. prop.test function in R is used to test whether male has significantly more DEGs in aging process than female. The RNA-seq data is deposited at Gene Expression Omnibus, accession GSE113195.

## Supplementary Material

Supplementary Table S1

Supplementary Table S2

Supplementary Table S3

Supplementary Table S4

Supplementary Table S5

Supplementary Table S6

Supplementary Table S7

Supplementary Table S8

Supplementary Table S9
